# The role of protein quality and amino acid composition in preventing sarcopenia and functional decline in older adults

**DOI:** 10.3389/fnut.2026.1817891

**Published:** 2026-05-08

**Authors:** Paula Calderón, Dolores Jima Gavilanes, Ana Sofía Vivanco-Zárate, Karen P. Sarango-González

**Affiliations:** 1 Nutrition and Dietetics Program, Faculty of Health Sciences, Technical University of Loja, Loja, Ecuador; 2 Escuela de Medicina, Universidad Espíritu Santo, Samborondón, Ecuador; 3 Medicine Program, Faculty of Health Sciences, Specialization in Imaging, College of Health Sciences, San Francisco University of Quito, Quito, Ecuador

**Keywords:** functional impairment, older adults, physiology of aging, protein quality, sarcopenia

## Abstract

Sarcopenia is a prevalent condition in older adults that involves the progressive loss of muscle mass and function, affecting their autonomy and quality of life. Balanced nutrition, especially adequate protein intake, is essential for preventing and managing this condition. This narrative review analyzes the importance of both the quantity and quality of protein in the diet of older adults to preserve muscle mass and prevent age-related functional decline. It is emphasized that general protein recommendations may be insufficient for this population, requiring higher doses of protein, appropriately distributed throughout the day, to optimally stimulate muscle protein synthesis. It also highlights the importance of the essential amino acid profile, particularly leucine, which plays a key role in activating anabolic pathways and improves the muscular anabolic response. Animal proteins offer greater bioavailability and a more complete amino acid profile, although strategic combinations of plant proteins can also be effective in meeting amino acid requirements. Other dietary supplements such as creatine, vitamin D, collagen, and omega-3 fatty acids may further complement nutritional interventions to improve muscle mass and function. These nutritional approaches, along with physical activity, particularly resistance training, form a comprehensive strategy for maintaining muscle function and overall wellbeing in older adults.

## 1 Introduction

Sarcopenia is a condition characterized by the progressive and generalized loss of muscle mass and function that primarily affects the older adult population, compromising their autonomy and quality of life, as it associated with a significantly increased risk of falls, hospitalizations, and mortality (1). This is due to age-related mechanisms of cellular senescence, oxidative stress, and low-grade chronic inflammation, all factors that exacerbate muscle catabolism and limit the regenerative capacity of the muscle tissue during aging (2). One of the cornerstones of the prevention and treatment of sarcopenia is a protein-rich diet; however, current evidence suggests that standard protein intake recommendations may be insufficient to overcome the “anabolic resistance” characteristic of advanced age (3, 4). This review objectively addresses protein intake in terms of quantity, daily distribution, and protein quality, highlighting the critical role of essential amino acids, such as leucine, and the potential benefits of strategic supplementation with creatine, vitamin D, and omega-3 fatty acids to promote healthy and functional aging.

## 2 Physiology of aging

**Figure 1:**
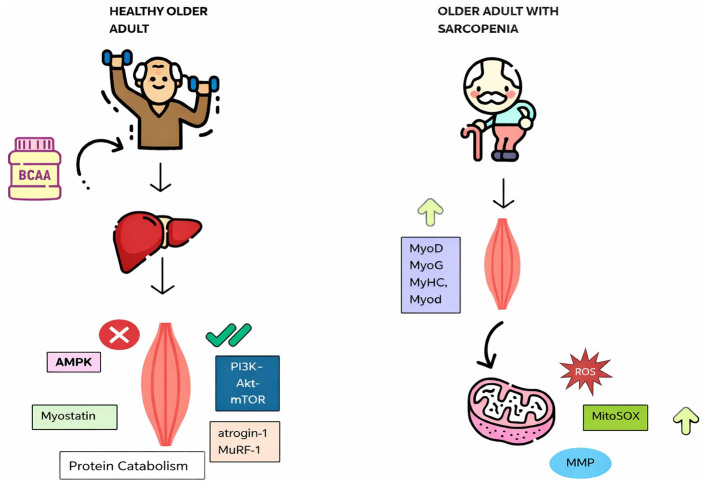
Physiology of aging. Key molecular mechanisms involved in the cellular aging process: loss of proteostasis, telomere shortening, epigenetic modifications, mitochondrial dysfunction, cellular senescence, and genomic instability. These processes interact dynamically and progressively, contributing to age-related functional decline (1, 2).

Cellular senescence, a state of irreversible cell cycle arrest, plays a crucial role in human aging and in the development of age-related diseases (5, 6). It is triggered by various stress factors, including oxidative stress, telomere shortening, and oncogene activation (6, 7). Oncogenes can be activated in response to abnormal cell proliferation signals and deoxyribonucleic acid (DNA) damage (6). The main molecular mechanisms, mediated by NF- κB, mTOR, and TGF- β, involved in cellular senescence are associated with the activation of transcriptional programs that regulate gene expression, signaling pathways, and metabolism regulators, developing a senescence-associated secretory phenotype (SASP) (5, 7). SASP is a group of inflammatory molecules, proteases, and growth factors secreted in response to cell damage and irreversible cell cycle arrest (5, 8) (Figure 1).

As we age, there is an increase in oxidative stress due to an imbalance between the production of reactive oxygen species (ROS) and the body's antioxidant defense mechanisms. The accumulation of ROS damages key cellular structures, such as DNA, proteins, and lipids, further promoting the onset of cellular senescence. Furthermore, dysfunction of protective signaling pathways such as hypoxia-inducible factor 1-alpha (HIF-1α) has been linked to the development of senescent phenotypes, especially in endothelial cells, and to the progression of cardiovascular diseases such as atherosclerosis (6, 9).

ROS plays a pivotal role in the induction and maintenance of cellular senescence, particularly through epigenetic mechanisms (10, 11). In turn, cellular senescence compromises tissue repair and regeneration, contributing to aging (5). With aging, muscle cells develop dysfunctional mitochondria (12), which leads to increased ROS production, perpetuating a cycle of oxidative damage, inflammation, and muscle atrophy, leading to decreased muscle strength and volume, which is associated with sarcopenia (13, 14). The selective elimination of senescent cells has emerged as a promising strategy to delay aging and mitigate age-related diseases (6, 10).

## 3 Sarcopenia in the elderly

Sarcopenia is a condition characterized by a gradual and extended decrease in muscle mass, associated with a reduction in strength and/or physical performance (15). According to the Asian Working Group on Sarcopenia, this condition is closely linked to aging, being more prevalent in older adults due to the physiological changes associated with this stage of life (16). With age, there is a reduction in type II muscle fibers and an increase in muscle fibrosis, which contributes to functional loss. In addition, several factors such as tobacco and alcohol use that negatively affect protein catabolism and living alone have been associated with an increased risk of developing sarcopenia (17, 18).

In this context, Stoodley et al. (3) point out that there is currently no approved pharmacological treatment for sarcopenia. They also highlight that inadequate dietary intake, especially of proteins, and a sedentary lifestyle are well-established contributors to this condition. Likewise, Choi and Kim (19) indicate that non-pharmacological methods for preventing sarcopenia include protein intake above the recommended levels, adequate intake of essential amino acids (EAA), and regular resistance exercise.

The diagnosis of sarcopenia is based on the updated criteria proposed in 2019 by the European Working on Sarcopenia in Older People (EWGSOP), which classify the condition into three progressive stages (3, 20, 21). The first stage, defined as probable sarcopenia, is identified by the presence of reduced muscle strength. This can be assessed through handgrip strength, with values below 27 kg in men and 16 kg in women, or by a time longer than 15 s in the five-times chair-rise test. The second stage, confirmed sarcopenia, is diagnosed when low muscle strength is accompanied by a reduction in muscle mass. Muscle mass is commonly measured using dual-energy X-ray absorptiometry (DEXA) and expressed as the height-adjusted Appendicular Skeletal Muscle Mass Index (ASMMI), with cut-off values below 7.0 kg/m^2^ in men and 6.0 kg/m^2^ in women. The most advanced stage, severe sarcopenia, is characterized by the presence of both reduced muscle strength and low muscle mass, together with impaired physical performance. Physical performance can be evaluated using functional tests such as the Short Physical Performance Battery (SPPB), with scores ≤ 8, or the Timed Up and Go (TUG) test, with times ≥ 20 seconds (21).

**Figure 2:**
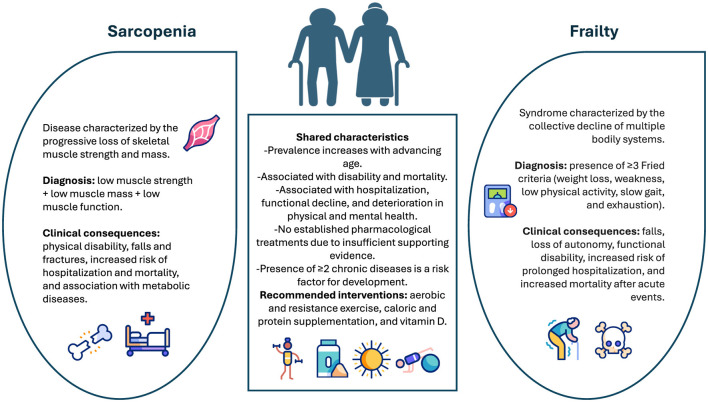
Sarcopenia and frailty: overlapping syndromes in older adults. The figure illustrates the key common and distinct features present in sarcopenia and frailty, two common conditions in older adults that share several factors and clinical effects. Sarcopenia is characterized by a gradual decline in strength, muscle mass, and muscle performance, while frailty is marked by a weakening of multiple systems that impacts independence. Both conditions are associated with disability, hospitalization, and mortality, and their management includes non-pharmacological interventions such as exercise and nutritional supplementation. This information is based on the studies by Thompson (20) and Ye et al. (122).

Individuals with sarcopenia are more vulnerable to adverse health outcomes including mobility impairment, higher risk of falls and fractures, and the onset or progression of chronic diseases (1). This condition has also been associated with the development of frailty syndrome, particularly the Fried frailty phenotype (FFP), which includes exhaustion, weakness, slowness, physical inactivity, and weight loss as diagnostic criteria (22) (Figure 2). A recent study conducted among older adults in China confirms this association, showing that sarcopenia is significantly and independently related to frailty and pre-frailty (23). Additionally, sarcopenia contributes to the progressive loss of functional autonomy and dependency, significantly affecting the quality of life (24). These complications result in an increase in hospitalizations, greater morbidity and mortality, as well as high costs for the health system due to the need for prolonged care and intensive rehabilitation (25, 26).

## 4 Nutrition in the elderly

Nutritional challenges for older adults include a progressive decline in energy requirements and food intake, which can lead to insufficient nutrient intake (27), and consequently may result in decline in physical and mental health as well as overall quality of life (28, 29).

Age-related physiological changes may further contribute to these challenges (30). Advancing age is associated with alterations that can negatively affect dietary intake and nutrient metabolism, including reduced appetite (the so-called “anorexia of aging”), changes in taste and smell perception, delayed gastric emptying, and other gastrointestinal alterations that may impair digestion and nutrient absorption (31). These physiological modifications, together with psychosocial and functional factors, can increase the risk of inadequate nutrient intake in older adults and contribute to the development of malnutrition (32).

Nutritional interventions for older adults focus on maintaining muscle mass and reducing malnutrition, often involving a multimodal approach, combining nutritional support with exercise, especially for frail and sarcopenic individuals (33, 34). Older adults require adequate energy intake to prevent unintentional weight loss. Recommendations for fat, carbohydrates, and fiber for this population are generally comparable to those for the general adult population, with an emphasis on complex carbohydrates, soluble and insoluble dietary fiber (25–35 g/day), and omega-3, 6, and 9 unsaturated fatty acids to promote metabolic health and prevent constipation (34, 35). Similarly, a relatively higher protein intake is recommended to prevent sarcopenia, as an inadequate protein intake has been associated with an increased risk of functional decline and malnutrition (36–40). In line adherence to balanced diets, such as Mediterranean or plant-based dietary patterns, rich in fruits, vegetables, whole grains, legumes and nuts, has been associated with better cognitive, physical, and cardiometabolic outcomes in older adults (41). Diet diversity also contributes to higher daily micronutrient intake, increasing the likelihood of meeting the Estimated Average Requirement (EAR) for key vitamins and minerals in this population (Table 1), based on the evidence reported in the literature (42–44). Moderate consumption of healthy animal-based foods such as low-fat dairy products and fish, and adequate fluids intake (1.6 liters/day for women and 2.0 liters/day for men) is generally recommended, with limiting the intake of red and processed meats, trans fats such as margarine and other ultra-processed products, and sugary beverages (42, 45).

**Table 1:** Estimated average requirements (EAR) for selected micronutrients in older adults: evidence from the literature.

Micronutrient	EAR Male	EAR Female
Minerals		
Calcium	800–1,100 mg	1,000–1,100 mg
Magnesium	350 mg	265 mg
Selenium	35–45 ug	30–45 ug
Vitamins		
Vitamin D	10–13 ug	10 ug
Thiamine	1.0–1.2 mg	0.9 mg
Riboflavin	1.1–1.4 mg	0.9–1.1 mg
Vitamin B12	1.4–3 μg	1.4–2 μg
Folate	200–320 μg	200–320 μg
Vitamin A	600–625 ug	500 ug
Vitamin C	60–75 mg	50–60 mg

Reported values of mineral and vitamin intake in older adults based on data from published studies (42–44).

Dietary interventions should be optimally personalized according to existing comorbidities and the risk of sarcopenia. A systematic review of 37 observational studies examined habitual micronutrient intake in more than 28,000 older adults (≥65 years) living independently in Western countries, with the aim of identifying the prevalence of inadequate intakes using the EAR cutoff method (46). Six micronutrients with a high prevalence of deficiency were identified: vitamin D (more than 84% of men and 91% of women), thiamin, riboflavin, calcium, magnesium, and selenium, highlighting these nutrients as priorities for public health interventions. Factors such as bioavailability, suboptimal supplement use, limitations in dietary assessment methods, and variability among nutritional recommendations affect the interpretation of these findings. Vitamin D emerges as particularly critical, despite fortification and supplementation, due to its relevance to bone, muscle, and possibly cognitive health. Likewise, deficiencies in calcium and magnesium have been associated with an increased risk of osteoporosis and sarcopenia, while B vitamins and certain antioxidants (selenium, vitamins A, C) often show inadequate intakes levels that may increase the risk of frailty, anemia and chronic diseases. Although supplement use is common, especially among older women, many individuals still fail to meet nutritional requirements (42).

Barriers to meeting the nutritional needs of older adults may include difficulties with food access and preparation (27). Importantly, nutritional counseling interventions that promote active engagement and individualized dietary guidance have shown promise in improving nutrition-related outcomes (47).

## 5 Protein consumption in older adults: quantity and quality

### 5.1 Amount of protein

Insufficient protein intake has been associated with the development of sarcopenia in both men and women particularly in older populations because the rates of protein synthesis and degradation in skeletal muscle are influenced by a complex interplay of various physiological, pathological, and nutritional factors, such as muscle contractility, systemic inflammation, stress, insulin sensitivity, and imbalanced dietary intake of energy, protein, amino acids, carbohydrates, lipids, vitamins, and minerals. In addition, aging is characterized by a phenomenon known as “anabolic resistance,” in which skeletal muscle shows a reduced sensitivity to the anabolic stimulus of dietary protein and physical activity (48, 49). This impaired anabolic response contributes to the progressive decline in muscle mass and strength observed with advancing age. Therefore, maintaining adequate and well-balanced nutrition is essential to preserve skeletal muscle mass and function in older adults, which in turn supports the maintenance of mobility and functional independence (50).

Furthermore, adequate protein intake becomes particularly important in the context of age-related changes in body composition, characterized by a progressive loss of lean mass and a relative increase in fat mass (51). In particular, a total protein intake below the Recommended Daily Intake (RDA) may adversely affect skeletal muscle size and function, as well as contribute to reductions in lean body mass among healthy older adults. On the other hand, protein consumption above the RDA has been associated with beneficial effects on the preservation of lean body mass and improvements in muscle strength and physical performance (52).

Both the World Health Organization (WHO) and the European Food Safety Authority (EFSA) recommend a Population Reference Intake (PRI) of 0.83 g of protein per kg of body weight per day, applicable to all adults regardless of age. Similarly, the U.S. Institute of Medicine proposed a similar RDA of 0.8 g/kg/day; however, these recommendations are primarily intended to prevent protein deficiency rather than to optimize musculoskeletal health in older adults. Consequently, several researchers have argued that these values may underestimate the actual protein requirements of older individuals, especially under conditions of physiological stress, chronic disease, or accelerated muscle loss (50, 53).

Studies have shown that older adults require higher protein doses per meal to optimally stimulate muscle protein synthesis compared to younger adults. While 0.24 g/kg of body weight per meal is sufficient in young adults, approximately 0.4 g/kg of body weight per meal is required in older individuals to achieve a comparable anabolic response (53). This difference reflects the reduced efficiency of protein turnover and synthesis in aging skeletal muscle. For this reason, not only balance in total daily protein intake but also the distribution of protein across meals have been suggested as an important strategy to optimize muscle protein synthesis in older adults.

**Figure 3:**
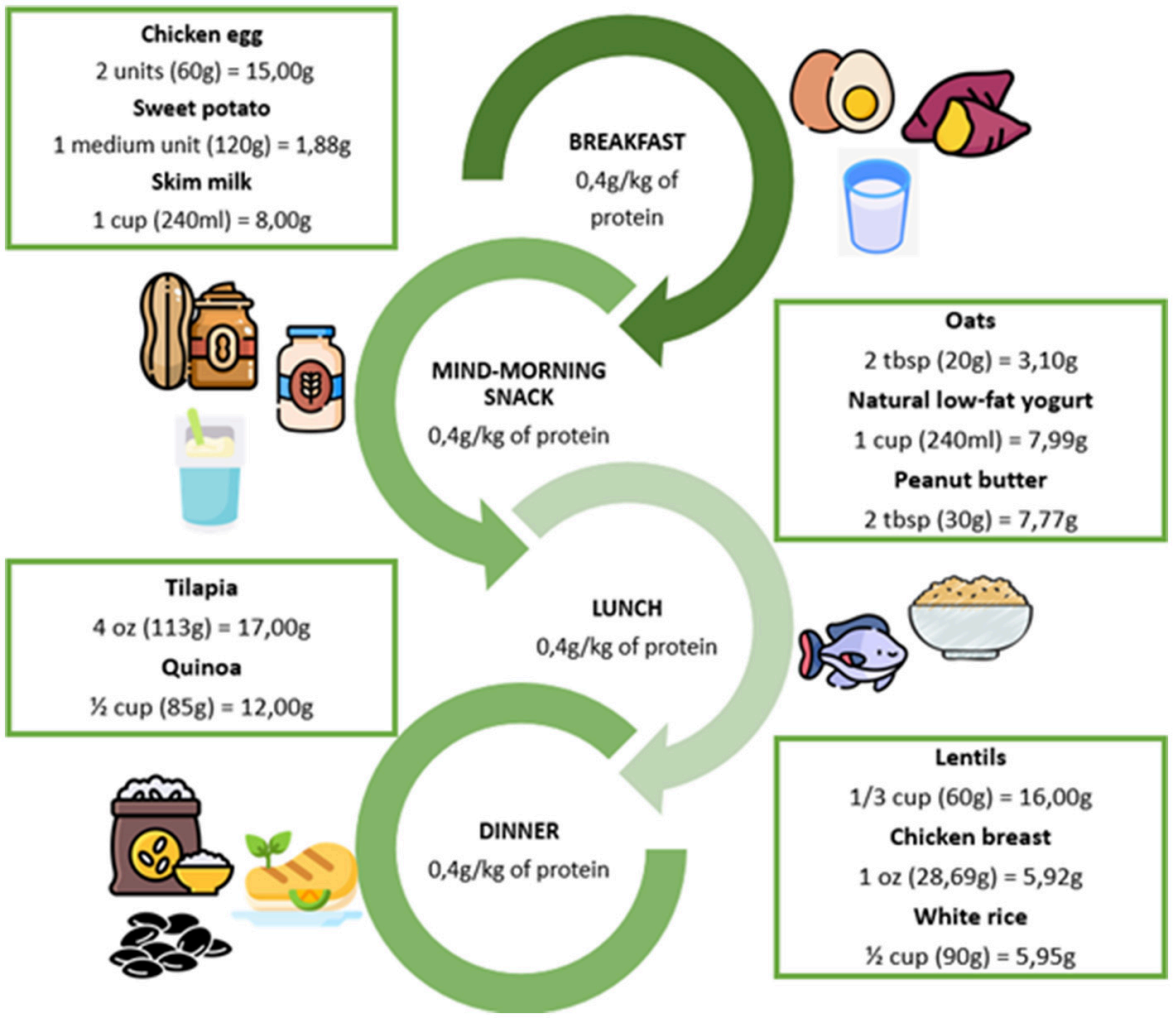
Distribution of daily protein requirement in a 70-kg older adult (1.2 g/kg/day). All foods were considered raw. Protein values were obtained from the chemical composition table of foods: based on nutrients of interest for the Ecuadorian population (52). Food icons were sourced from Flaticon.

**Figure 4:**
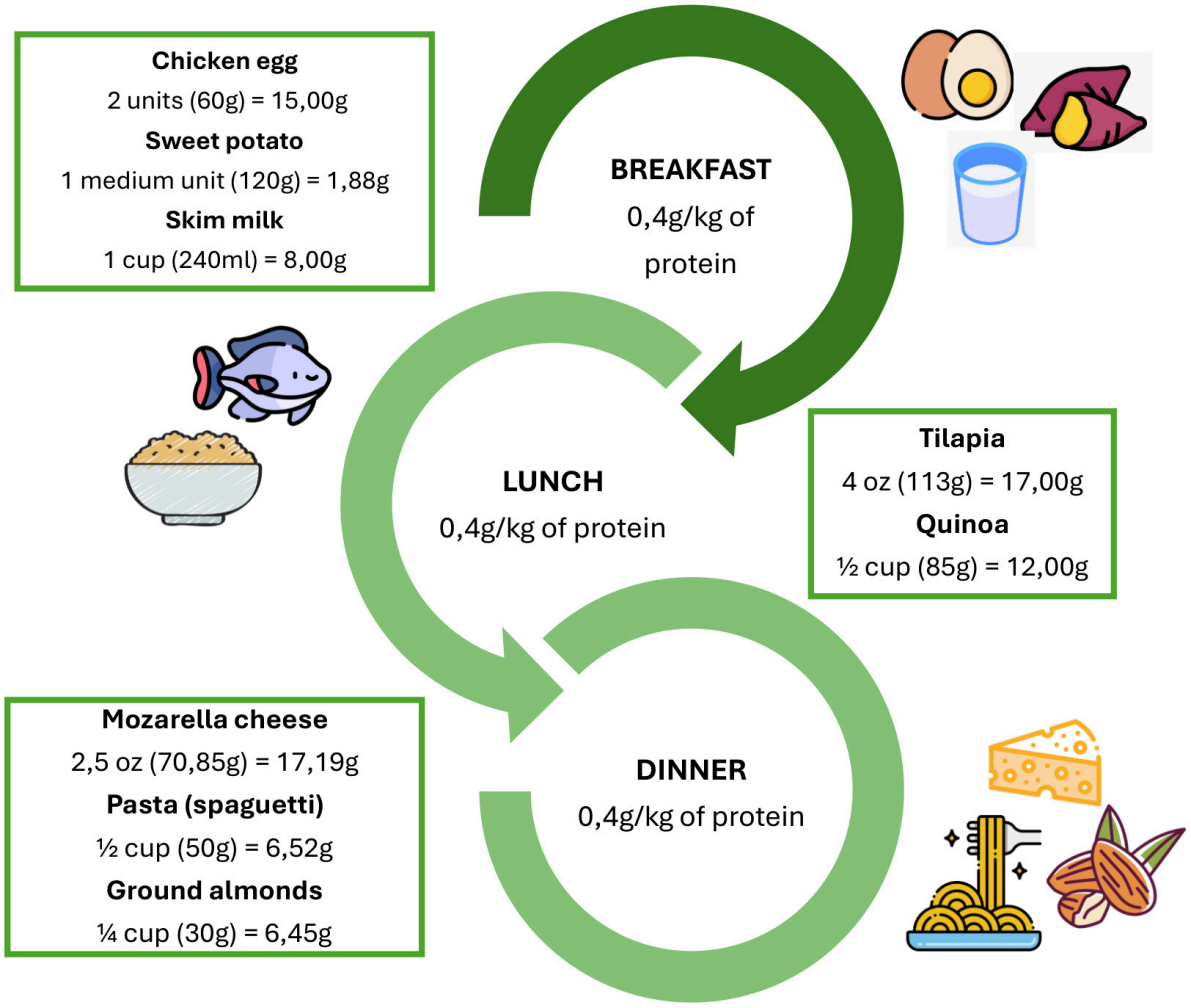
Distribution of daily protein requirement in a 70-kg older adult (1.5 g/kg/day). All foods were considered raw. Protein values were obtained from the chemical composition table of foods: based on nutrients of interest for the Ecuadorian population (53) Food icons were sourced from Flaticon.

Several organizations and expert groups, including the European Society for Clinical Nutrition and Metabolism (ESPEN), recommend a daily protein intake of 1.0–1.2 g/kg of body weight for healthy older adults, while in the presence of acute or chronic disease, this amount may be increased to approximately 1.2–1.5 g/kg/day, and in cases of severe illness, trauma, or malnutrition, intakes of up to 2.0 g/kg/day may be considered (38–40) (Figure 3). Some evidence also suggests that dietary patterns providing approximately 1.5 g/kg/day of protein may further enhance muscle protein synthesis and contribute to the preservation of muscle mass in older adults (53) (Figure 4).

### 5.2 Protein quality: essential amino acids and bioavailability

Dietary proteins serve not only as a source of energy and nitrogen, but also provide the amino acids necessary for protein synthesis in the human body. Of the 20 amino acids involved in this process, nine are considered essential (histidine, isoleucine, leucine, lysine, methionine, phenylalanine, threonine, tryptophan and valine), as they cannot be synthesized endogenously and therefore must be introduced through the diet (54).

Adequate availability of essential amino acids (EAAs) is particularly important in older adults, as age-related anabolic resistance increases the requirement for high-quality protein sources to effectively stimulate muscle protein synthesis (55). Beyond the amount consumed, the quality of dietary protein represents a key factor in preventing sarcopenia. This quality is primarily determined by the EAAs profile and protein digestibility. EAAs not only act as building blocks for muscle tissue but also function as metabolic signals capable of activating intracellular pathways involved in protein synthesis (55).

In this sense, animal proteins are generally considered more effective in stimulating muscle protein synthesis, as they contain the nine EAAs in proportions that closely match human physiological requirements and typically exhibit high digestibility, which results in greater postprandial plasma amino acid availability and a stronger anabolic response (56). These characteristics have been associated with improved preservation of muscle mass, reduced risk of frailty, and attenuation of age-related declines in muscle strength in older adults (53).

In contrast, many plant proteins lack one or more key EAAs, such as lysine or methionine, and their digestibility and bioavailability may be reduced due to the presence of antinutritional factors and plant cell wall structures, potentially limiting their anabolic capacity if not properly combined or consumed in sufficient quantities (53, 56). Nevertheless, both animal and plant protein sources can contribute to meeting essential amino acid requirements when appropriately incorporated into a balanced dietary pattern, such as plant-based diet that is more sustainable for health and for the environment (57, 58). However, animal proteins generally contain higher concentrations of leucine, a key amino acid involved in activating the mechanistic target of rapamycin (mTOR) signaling pathway, which plays a central role in initiating muscle protein synthesis and promoting muscle anabolism (59). This difference in the essential amino acid composition between plant and animal protein sources is evident in Table 2, which shows the specific essential amino acid content in representative foods from both groups. These compositional differences help explain the variability in anabolic responses observed following the consumption of different protein sources.

**Table 2:** Essential amino acid content of plant-based and animal-based protein foods.

	Content of essential amino acids (EAAs) in the food								
Food	Histidine (g)	Isoleucine (g)	Leucine (g)	Lysine (g)	Methio-nine (g)	Phenyla-lanine (g)	Threo-nine (g)	Trypto-phan (g)	Valine (g)
Plant-based foods									
White rice	0.155	0.285	0.546	0.239	0.155	0.353	0.236	0.077	0.403
Green pea	0.107	0.195	0.323	0.317	0.082	0.200	0.203	0.361	0.235
Rolled oats, precooked	0.405	0.694	1.284	0.701	0.312	0.895	0.575	0.234	0.937
Sweet potato	0.031	0.055	0.092	0.066	0.029	0.089	0.203	0.130	0.086
Quinoa	0.407	0.504	0.840	0.766	0.309	0.593	0.421	0.16	0.594
Green fava bean	0.134	0.251	0.432	0.366	0.043	0.228	0.208	0.056	0.274
Red kidney bean	0.650	1.040	1.880	1.610	0.350	1.270	0.990	0.270	1.230
Chickpea	0.566	0.882	1.465	1.377	0.270	1.103	0.766	0.200	0.865
Soybean	1.097	1.971	3.309	2.706	0.547	2.122	1.766	0.591	2.029
Animal-based foods									
Shrimp	0.485	0.974	1.681	1.813	0.557	0.946	0.824	0.186	0.935
Croaker	0.523	0.819	1.445	1.633	0.526	0.694	0.780	0.199	0.916
Pork, lean meat	0.856	1.003	1.719	1.927	0.567	0.855	0.978	0.272	1.162
Turkey breast, skinless	0.776	1.131	1.741	2.061	0.608	0.944	1.078	0.296	1.059
Chicken, whole with skin	0.544	0.924	1.350	1.509	0.493	0.721	0.767	0.207	0.902
Beef, lean meat	0.890	1.118	2.063	2.305	0.637	0.955	1.127	0.269	1.182
Beef liver	0.629	0.961	1.910	1.607	0.543	1.084	0.869	0.263	1.260
Whole cow's milk	0.089	0.198	0.321	0.260	0.082	0.158	0.148	0.046	0.220
Fresh cheese, semi-hard, semi-fat	0.545	1.157	1.875	1.061	0.544	1.034	0.989	0.503	1.361
Whole chicken egg	0.309	0.671	1.086	0.912	0.380	0.680	0.556	0.167	0.858

All foods were analyzed in their raw state, and values are presented per 100 grams of edible portion. This is important to consider, as amino acid content may be altered by cooking or processing. adapted from: Colombian food composition table, 2018 (125).

Recent research has shown that certain combinations of plant proteins or the mixture of animal and plant protein sources can generate comparable muscle protein synthesis responses, especially in young adults, which is attributed to a more balanced amino acid profile and a more sustained release of amino acids into the circulation (50). However, in older adults, whose anabolic response is less efficient, an optimized intake of both quantity and quality becomes particularly important. Therefore, strategically combining animal and plant protein sources within the diet may represent an effective nutritional strategy to prevent or delay sarcopenia and age-related functional decline (50, 52). A recent meta-analysis including 43 randomized controlled trials compared the effects of plant and animal protein on muscle health. Of these, 30 studies were included in the quantitative analysis. The results showed that plant protein was associated with slightly lower gains in muscle mass compared with animal protein (SMD = −0.20; 95% CI: −0.37–0.03), with stronger differences observed in younger adults (< 60 years) and minimal differences in older adults (≥60 years). No significant differences were found between plant and animal protein regarding muscle strength or physical performance. Interestingly, soy protein showed comparable effects to milk protein on muscle mass, whereas animal protein appeared to have a modest advantage over other non-soy plant proteins such as rice, oat, potato, and chia. Overall, the findings suggest that animal protein may confer a small benefit for muscle mass compared with some plant proteins, although further research on a wider range of plant protein sources and dietary patterns is needed (58, 60).

A deficiency in a single amino acid (the so-called limiting amino acid) can compromise the effective utilization of all ingested protein, since the body does not possess a storage system for amino acids comparable to that for carbohydrates or lipids; their availability therefore depends largely on regular dietary intake (61). This aspect is particularly relevant in older adults, whose protein requirements are increased while appetite and food intake may often decline. Therefore, nutritional strategies targeting this population should focus not only on increasing total protein intake but also on optimizing protein quality, prioritizing sources with a balanced amino acid composition and high bioavailability (62).

In addition to considering the quantity and quality of proteins consumed, circulating amino acid profile has recently emerged as a potentially valuable biomarker for evaluating skeletal muscle status in older adults, since muscle acts as the primary reservoir of amino acids during the post-absorptive state, with circulating levels that partially reflect muscle mass and metabolic activity. This profile has been shown to vary in older individuals with conditions such as obesity, sarcopenia, physical frailty, and insulin resistance, highlighting metabolic alterations associated with aging and muscle deterioration (63, 64). Moreover, emerging evidence suggests that amino acids may play an additional role in modulating gut microbiota composition and activity. Some researchers have proposed the concept of “aminobiotics,” referring to amino acids that can be metabolized by specific intestinal bacteria, thereby supporting host–microbiome interactions and potentially contributing to metabolic health. Although this field is still developing, these findings suggest a possible link between protein nutrition, gut microbiota, and musculoskeletal health in aging populations (65).

### 5.3 Key amino acids and supplementation in the prevention and treatment of sarcopenia

Aging muscle requires higher amounts of amino acids (AA) to stimulate muscle anabolism due to a reduced efficiency of molecular pathways involved in muscle protein synthesis in response to hyperaminoacidemia, a phenomenon commonly referred to as anabolic resistance. If this resistance is not compensated by a proportionally higher protein and amino acid intake, muscle protein synthesis may decline further, generating an imbalance in muscle metabolism that favors the decline of protein turnover that favors proteolysis and progressive muscular atrophy (66).

Among the amino acids involved in the regulation of skeletal muscle metabolism, branched-chain amino acids (BCAAs) have received particular attention. BCAAs, valine, isoleucine and leucine, are essential organic compounds that play a crucial role in regulating different processes such as energy homeostasis, protein synthesis and metabolism, by stimulating muscle protein synthesis through the activation of the mTOR pathway (67–71). In addition to mTOR signaling, other molecular pathways contribute to the regulation of skeletal muscle mass. For example, the β-catenin signaling pathway has been proposed as an alternative regulatory mechanism involved in muscle growth and regeneration (67).

Several studies focused on the relationship between BCAAs and musculoskeletal outcomes. A recent study by Bai et al. showed positive associations between circulating BCAA concentrations and skeletal muscle mass and suggested that muscle mass may partially mediate the relationship between BCAAs and muscle strength, particularly when combined with other nutrients such as vitamin D in older adults and individuals with sarcopenia (67, 72). Additionally, it has been suggested that BCAAs may contribute to the inhibition of muscle protein degradation by downregulating components of the ubiquitin–proteasome system (UPS), including the E3 ubiquitin ligases atrogin-1 and MuRF-1, which are key regulators of muscle protein breakdown (73). Furthermore, clinical and translational studies suggest that BCAA supplementation at doses exceeding approximately 0.20g/kg/day may lead to measurable improvements in muscle mass and strength, as well as functional outcomes such as gait speed and chair-rise performance in older adults, particularly when combined with vitamin D supplementation (67–69, 72, 74).

**Figure 5:**
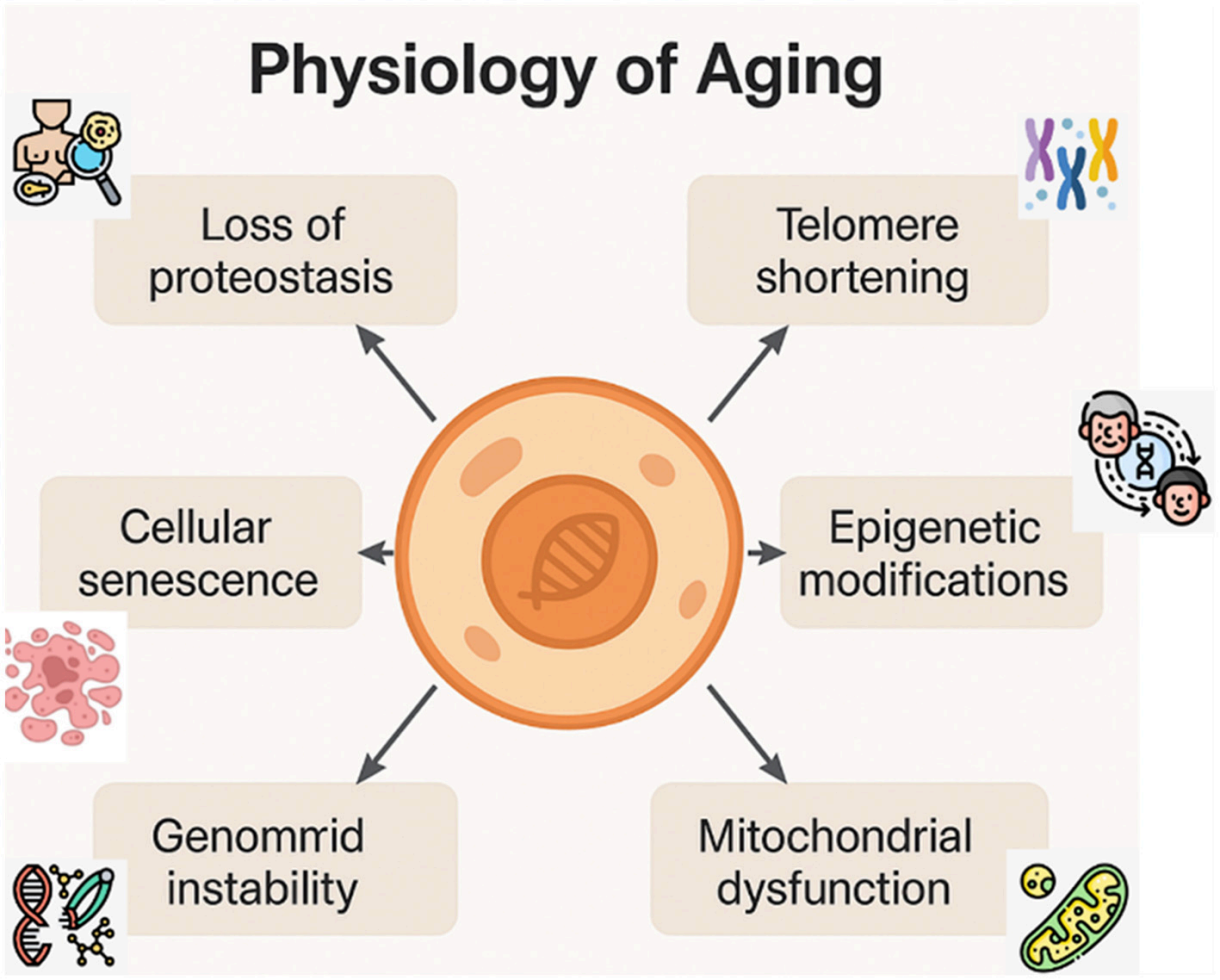
Molecular activation between a healthy older adult and an older adult with sarcopenia. In healthy adults, it is evident that after the consumption of amino acids in conjunction with exercise, they activate the PI3K / Akt / mTOR signaling pathway and also the alternative pathways of the ubiquitin-proteasome system, inhibiting the expression levels of MuRF1 and Atrogin-1, which inhibits protein catabolism. While in sarcopenia the PI3K / Akt / mTOR signaling pathway is inactivated, the expression levels of MyoD (myogenin), MyoG and MyHC (myosin heavy chains) are promoted, generating muscle atrophy. Additionally, mitochondrial dysfunction is increased by inhibiting the production of ROS and MitoSOX and increasing the mitochondrial membrane potential (123, 124).

The main molecular mechanisms and signaling pathways involved in skeletal muscle aging and sarcopenia are summarized in Figure 5. At the molecular level, leucine acts as a substrate and signaling molecule. It is detected by specific intracellular sensors, such as leucyl -tRNA synthetase and sestrin 2, which facilitate the activation of the Rag GTPases (50, 73, 75). These molecular events promote the translocation and activation of the mTOR complex 1 (mTORC1) at the lysosomal surface, leading to the phosphorylation of downstream effectors involved in the initiation of mRNA translation and muscle protein synthesis in skeletal muscle (76). Supporting this mechanism, Casperson et al. showed that a 4 g leucine/meal supplementation with three main meals improved muscle protein synthesis (50, 76). In parallel with anabolic pathways, several catabolic pathways regulate muscle protein degradation during aging. The autophagy-lysosomal system contributes to muscle protein degradation in sarcopenia and plays an essential role in the removal of damaged organelles and long-lived proteins, functioning in coordination with the ubiquitin–proteasome system. Certain bioactive compounds, such as docosahexaenoic acid (DHA), have been shown to modulate both the UPS and the autophagy-lysosomal system, potentially attenuating muscle loss by reducing excessive proteolysis and promoting cellular homeostasis (77).

Among other amino acids playing an important role in muscle functioning, methionine is has been shown to be involved in the synthesis of S-adenosyl-L-methionine (SAM), a key metabolite for the regulation of autophagy in muscle cells. In preclinical studies, betaine in conjunction with methionine has been reported to improve the level of autophagy in aged skeletal muscle through the Mettl21c/p97/VCP axis to delay muscle loss (78). Likewise, tryptophan plays multiple metabolic roles beyond protein synthesis, acting as a precursor for several metabolic pathways, including serotonin synthesis and the kynurenine pathway (79). Experimental evidence indicates that tryptophan can stimulate the expression of myogenic regulatory factors, such as myogenin, MyoD, and myosin heavy chain, in C2C12 myoblasts in vitro while also activating the IGF-1/p70S6K/mTOR signaling pathway in skeletal muscle (80). However, aging is frequently accompanied by a chronic low-grade inflammatory state, which activates indoleamine-2, 3-dioxygenase 1 (IDO1) and accelerates tryptophan depletion, leading to the accumulation of kynurenine and related metabolites. This metabolic shift has been associated with impaired tissue homeostasis, reduced protein synthesis, and progressive muscle atrophy (81, 82). In animal models, experimental tryptophan deficiency led to a decrease in muscle fiber diameter and muscle mass, effects that were reversible after restoration of tryptophan in the diet. This, coupled with the anabolic products of this pathway, such as picolinic acid, has been shown to have a beneficial impact on skeletal muscle and bone tissue (83). Physical exercise modulates the kynurenine pathway by enhancing the conversion of kynurenine into kynurenic acid and other anabolic metabolites in skeletal muscle, which may contribute to improved muscle function and protection against age-related musculoskeletal decline (79). In addition to general physical activity, resistance training plays a particularly important role in skeletal muscle health, as it represents the main stimulus for muscle protein synthesis, hypertrophy, and strength maintenance during aging, and is therefore considered a key strategy for the prevention and treatment of sarcopenia (84). Another amino acid that has attracted growing interest in the context of aging and muscle metabolism is L-glutamine. L-glutamine supports metabolic health through several interconnected mechanisms, including the regulation of lipid and glucose metabolism and the suppression of hepatic inflammation (85–87).

Skeletal muscle represents the main site of glutamine synthesis, in sarcopenia there is an alteration of protein and amino acid metabolism, including the reduction of branched-chain amino acid levels and glutamine, which contributes to muscle wasting (88, 89). In adipose tissue and skeletal muscle, glutamine supplementation has been shown to restore intracellular levels of adenosine and glutathione, two key molecules involved in antioxidant defense and cellular energy balance (87, 90, 91). Furthermore, glutamine contributes to maintaining intestinal barrier integrity, promotes the growth of beneficial gut microbiota, and reduces the proliferation of potentially pathogenic bacteria, thereby improving nutrient absorption and lowering systemic inflammation. Experimental studies suggest that early glutamine supplementation may also prevent age-related intestinal atrophy by increasing intestinal mass and improving mucosal structure (86, 92). In older adults (≥60 years) with sarcopenia, some studies have suggested a glutamine supplementation dose of approximately 0.3 g/day, which, when combined with resistance exercise, may help counteract muscle loss and improve metabolic resilience (93).

### 5.4 Non-protein supplements key in the approach to sarcopenia

In addition to adequate protein intake, several non-protein nutritional supplements have shown positive effects in the prevention and treatment of sarcopenia. Compounds such as creatine, collagen, omega-3 fatty acids, and vitamin D may contribute to preserving muscle mass and function through various mechanisms because of their involvement in energy metabolism, muscle protein turnover, inflammation control, and neuromuscular function (94). In older adults, these supplements may potentially contribute to improving muscle mass, strength, functional performance, and overall quality of life when used as part of a multimodal intervention that includes adequate nutrition and regular physical activity (94, 95). The following sections summarize the physiological characteristics and potential benefits of these supplements in the context of sarcopenia, highlighting the mechanisms through which they may influence skeletal muscle metabolism and functional outcomes in aging populations. Suggested doses for preventive and therapeutic use based on the available clinical studies are presented in Table 3.

**Table 3:** Evidence from studies on non-protein supplementation for the prevention and treatment of sarcopenia in older adults.

	Prevention dose	Treatment dose
Creatine	1–3 g/day	>5 g/day
Collagen	>2.5 g/day	15 g/day (with physical activity) up to 30 g/day (low activity)
Vitamin D	700 to 1,000 IU/day	800 IU/12 h
Omega-3 fatty acids	1.2 g of EPA/day 1.2 g of DHA/day	1.86 g of EPA/day 1.5 g of DHA/day

The data obtained correspond to recommendations in clinical studies (100, 111, 126–128).

#### 5.4.1 Creatine

Creatine is a naturally occurring nitrogen-containing compound synthesized from the amino acids arginine, glycine, and methionine and is primarily stored in skeletal muscle (96). An important function of creatine in excitable tissues is to allow accelerated regeneration of ATP (adenosine triphosphate) during periods of increased energy demand, allowing maintenance of power and muscle contraction. This can be achieved through both temporal and spatial functions. The phosphorylated form of intracellular creatine acts as a readily available source of cellular phosphate, which when dephosphorylated releases a phosphate molecule that can be used to rephosphorylate ATP from ADP (adenosine diphosphate) (97). This rapid ATP buffering system is particularly important during short-duration, high-intensity muscle contractions, such as those involved in resistance exercise. In older adults, creatine supplementation has been proposed as a potential strategy to counteract age-related declines in muscle mass and strength, especially when combined with resistance training. Recent reviews also suggest that creatine may contribute to improved muscle function, reduced inflammation, and a lower risk of falls in aging population (98). Liu et al. (99) suggested a dose between 1 and 3 g/day to maintain normal creatine stores and obtain free energy from catabolism, depending on muscle mass. Forbes et al. (100) concluded that older adults aiming to improve whole-body lean tissue mass and strength may benefit from supplementation of 5 grams daily, although older adults aiming to improve functionality, posture, or the ability to perform upper-body activities may need a lower dose, less than 5 grams per day, taking into account that supplementation should be given within 60 min after resistance training, as exercise enhances creatine uptake. Furthermore, it has been shown that creatine supplementation of more than 5g/day for a period of 24 months increases intramuscular glycogen storage, increasing muscle strength, especially in the lower limbs (100). Creatine supplementation seems to improve the adaptive response of muscles to training, as it increases the capacity to exercise at high intensities and promotes post-exercise recovery and adaptation (100). These musculoskeletal benefits support its use as a potential intervention in the treatment of frailty and cachexia (100, 101).

#### 5.4.2 Collagen

Collagen is the most abundant structural protein in the human body and is a key component of connective tissues such as tendons, ligaments, skin, and cartilage (102). In the context of musculoskeletal aging, collagen supplementation has attracted interest because of its potential effects on connective tissue integrity, joint health, and muscle function, through mechanisms related to connective tissue remodeling and improved recovery following physical exercise (103, 104). The collagen molecule is composed of a repeating sequence of three amino acids (glycine, proline, hydroxyproline). In general, collagen contains approximately 30% glycine, 12% proline, 11% alanine, 10% hydroxyproline, and 1% hydroxylysine (105).

Regarding the impact of collagen intake on body composition, Zdzieblik et al. (106) showed that older men with sarcopenia who exercised three times per week and consumed 15 g of collagen peptide daily for 12 weeks experienced significant body changes, with an average increase of 4.2 kg in fat-free mass, compared to only 2.9 kg in the placebo group. Conversely, regarding how collagen influences muscle protein production, Oikawa and colleagues administered 30 g of whey protein or collagen peptide twice daily to elderly individuals with limited physical activity and low energy, and only the whey protein group showed an increase in lean mass and muscle protein production in the lower extremities upon return to physical activity (107). These findings suggest that collagen supplementation may be more effective when combined with exercise and adequate intake of high-quality proteins providing essential amino acids.

#### 5.4.3 Vitamin D

Vitamin D is a fat-soluble hormone-like vitamin primarily synthesized in the skin through exposure to ultraviolet B radiation and obtained to a lesser extent through dietary sources (108). Beyond its well-known role in calcium and bone metabolism, vitamin D has emerged as an important regulator of skeletal muscle physiology (109). Vitamin D plays a crucial role in skeletal muscle health by regulating calcium metabolism, promoting protein synthesis, and modulating muscle cell proliferation and differentiation (110). It positively regulates the expression of muscle-specific genes, such as MyHC and muscle creatine kinase (MCK), leading to the differentiation and maturation of muscle cells (110). Several studies have explored the effect of vitamin D intervention in older people and its effects on age-related outcomes. The most significant effects were seen in people over 65 years of age with vitamin D levels below 30 nmol/L, who received supplementation of between 700 and 1,000 IU daily (111), resulting in improved mobility, suggesting that vitamin D supplementation helps maintain type II muscle fibers, which are crucial for maintaining strength and mobility, thereby reducing the risk of falls and increasing physical performance (110). However, a recent meta-analysis comprising seven randomized controlled trials and examining whether vitamin D supplementation influences body composition parameters in individuals aged ≥60 years did not report significant improvement in skeletal muscle mass index, lean mass, or muscle strength in individuals supplemented with vitamin D compared with control groups. A modest reduction in fat mass was observed in the pooled analysis; however, this effect appeared to be largely driven by a single study and lost significance when trials using active vitamin D were excluded. Consequently, the findings suggest that vitamin D intake alone is unlikely to produce clinically meaningful improvements in muscle-related body composition outcomes in older adults, although further well-designed trials are required to clarify its potential role (94, 95). Nonetheless, strategies to prevent vitamin D deficiency, such as foods fortification and its supplementation could potentially contribute to mitigating age-related muscle weakness when integrated into multimodal interventions (95, 112, 113). Widajanti et al. (114) suggested in their meta-analysis that vitamin D supplementation showed no effect on appendicular skeletal muscle mass, thus the treatment of sarcopenia should not only depend on supplementation with this vitamin but also on protein intake and support with physical exercise.

#### 5.4.4 Omega-3 fatty acids

Omega-3 polyunsaturated fatty acids (PUFAs), particularly eicosapentaenoic acid (EPA) and docosahexaenoic acid (DHA), are long-chain fatty acids commonly found in marine foods such as fatty fish. These compounds have been extensively studied for their anti-inflammatory and metabolic effects, which may be particularly relevant in the context of age-related muscle loss (115). PUFAs have been shown to reduce the expression of proinflammatory genes and modulate pathways associated with endoplasmic reticulum stress, autophagy and protein degradation, helping to preserve muscle mass, improve mitochondrial function and motor functionality (99, 116), and to regulated chronic low-grade inflammation, which is considered one of the key mechanisms contributing to sarcopenia (117).

Omega-3 supplementation at a dose of 2 g/d in older adults has shown beneficial effects, such as increased muscle mass, improvements in isometric strength, and an increase in gait speed, especially when the intervention is maintained for at least six months (99). A study by Yoshino et al. (118), in 20 older adults evaluated the impact of daily supplementation with 1.86 g of EPA and 1.50 g of DHA for 6 months, the results showed an increase of 10.3 ± 2.2% in thigh muscle volume, an improvement of 10.3 ± 3.3% in hand grip strength and an increase of 9.1 ± 4.1% in maximum muscle strength (1-RM), in addition to a reduction of 11.5 ± 5.2% in intermuscular fat content, also showed the increase in the expression of the *UQCRC1* and *UCP3 genes*, which are involved in mitochondrial biogenesis and regulation of muscle mass. Zhang et al. (119) evaluated the relationship between dietary levels of omega-3 and omega-6 polyunsaturated fatty acids (PUFAs) and sarcopenia, finding a significant inverse association between omega-3 consumption and the presence of sarcopenia, while no clear relationship was evident with omega-6 levels, these results reinforcing the possible importance of adequate omega-3 consumption in preventing sarcopenia. These findings collectively suggest that omega-3 fatty acids may represent a promising nutritional strategy to support muscle metabolism and functional capacity in aging populations, however, future research is needed to better understand their mechanism of action (120, 121).

## 6 Conclusion

Despite considerable progress in understanding the impact of protein quality and quantity on preventing sarcopenia and functional loss in older adults, significant challenges remain in improving nutritional strategies. Evidence suggests that older adults with sarcopenia require protein intake that exceeds general guidelines, focusing on high-quality proteins and a balanced profile of essential amino acids, with particular emphasis on leucine, to improve muscle protein synthesis. Furthermore, the inclusion of specific supplements such as creatine, vitamin D, and omega-3 fatty acids has shown potential positive effects on muscle function, increased muscle mass, and reduced inflammation. However, further studies are needed to identify the optimal doses and protein sources, and the most appropriate timing of protein intake. A multidisciplinary approach, combining personalized nutrition with physical activity, may prove to be the best strategy to maintain autonomy and improve quality of life in older adults, promoting healthy and functional aging.

## References

[1] Veronese N Demurtas J Soysal P Smith L Torbahn G Schoene D . Sarcopenia and health-related outcomes: an umbrella review of observational studies. Eur Geriatr Med. (2019) 10:853–62. doi: 10.1007/s41999-019-00233-w 34652767

[2] Aversa Z Zhang X Fielding RA Lanza I LeBrasseur NK. The clinical impact and biological mechanisms of skeletal muscle aging. Bone. (2019) 127:26–36. doi: 10.1016/j.bone.2019.05.021 31128290 PMC6708726

[3] Stoodley IL Berthon BS Scott HA Williams EJ Baines PJ Knox H . Protein intake and physical activity levels as determinants of sarcopenia risk in community-dwelling older adults. Nutrients. (2024) 16:9. doi: 10.3390/nu16091380 38732628 PMC11085115

[4] Paddon-Jones D Rasmussen BB. Dietary protein recommendations and the prevention of sarcopenia. Curr Opin Clin Nutr Metab Care. (2009) 12:86–90. doi: 10.1097/MCO.0b013e32831cef8b 19057193 PMC2760315

[5] Kumari R Jat P. Mechanisms of cellular senescence: cell cycle arrest and senescence associated secretory phenotype. Front Cell Dev Biol. (2021) 9:645593. doi: 10.3389/fcell.2021.645593 33855023 PMC8039141

[6] Marcozzi S Beltrami AP Malavolta M. Molecular mechanisms to target cellular senescence in aging and disease. Cells. (2022) 11:23. doi: 10.3390/cells11233732 36496992 PMC9737399

[7] Wei W Ji S. Cellular senescence: molecular mechanisms and pathogenicity. J Cell Physiol. (2018) 233:9121–35. doi: 10.1002/jcp.26956 30078211

[8] Andonian BJ Hippensteel JA Abuabara K Boyle EM Colbert JF Devinney MJ . Inflammation and aging-related disease: a transdisciplinary inflammaging framework. Geroscience. (2025) 47:515–42. doi: 10.1007/s11357-024-01364-0 39352664 PMC11872841

[9] Cassotta M Quiles J Giampieri F Battino M. Aging, Age-Related Diseases, Oxidative Stress and Plant Polyphenols: Is this a True Relationship?. Available online at: https://journals.sagepub.com/doi/full/10.3233/MNM-240057 (Accessed August 06, 2025).

[10] Davalli P Mitic T Caporali A Lauriola A D'Arca D. ROS, cell senescence, and novel molecular mechanisms in aging and age-related diseases. Oxid Med Cell Longev. (2016) 2016:3565127. doi: 10.1155/2016/3565127 27247702 PMC4877482

[11] Chajadine M Laurans L Radecke T Mouttoulingam N Al-Rifai R Bacquer E . Harnessing intestinal tryptophan catabolism to relieve atherosclerosis in mice. Nat Commun. (2024) 15:6390. doi: 10.1038/s41467-024-50807-x 39080345 PMC11289133

[12] Kim JE Kwon EY Han Y. A collagen hydrolysate containing tripeptides ameliorates sarcopenia in middle-aged mice. Molecules. (2022) 27:9. doi: 10.3390/molecules27092718 35566067 PMC9104253

[13] Zhang H Qi G Wang K Yang J Shen Y Yang X . Oxidative stress: roles in skeletal muscle atrophy. Biochem Pharmacol. (2023) 214:115664. doi: 10.1016/j.bcp.2023.115664 37331636

[14] Chapman J Fielder E Passos JF. Mitochondrial dysfunction and cell senescence: deciphering a complex relationship. FEBS Lett. (2019) 593:1566–79. doi: 10.1002/1873-3468.13498 31211858

[15] Chew STH Tey SL Yalawar M Liu Z Baggs G How CH . Prevalence and associated factors of sarcopenia in community-dwelling older adults at risk of malnutrition. BMC Geriatr. (2022) 22:997. doi: 10.1186/s12877-022-03704-1 36564733 PMC9789557

[16] Chen LK Woo J Assantachai P Auyeung TW Chou MY Iijima K . Asian working group for sarcopenia: 2019 consensus update on sarcopenia diagnosis and treatment. J Am Med Dir Assoc. (2020) 21:300–7.e2. doi: 10.1016/j.jamda.2019.12.012 32033882

[17] Guo Q Chen Q Chen K. Comparative analysis of SARC-F-EBM, Ishii test, and six other screening tools for sarcopenia in Chinese community-dwelling older adults: a cross-sectional diagnostic study. Sci Rep. (2024) 14:24679. doi: 10.1038/s41598-024-75975-0 39433943 PMC11494060

[18] Polo-Ferrero L Recio-Rodriguez JI González-Manzano S Sáez-Gutiérrez S Barbero-Iglesias FJ Méndez-Sánchez R. Prevalence and risk factors for sarcopenia in active community-dwelling older adults according to the EWGSOP2 criteria. Geriatric Nursing. (2024) 60:361–6. doi: 10.1016/j.gerinurse.2024.09.018 39393305

[19] Choi J-Y Kim K-I. Diagnosis and Management of Sarcopenia. Available online at: https://jkma.org/journal/view.php?doi=10.5124/jkma.2024.67.7.461 (Accessed August 05, 2025).

[20] Thompson C. Frailty and sarcopenia. Medicine. (2024) 52:652–5. doi: 10.1016/j.mpmed.2024.08.006

[21] Cruz-Jentoft AJ Bahat G Bauer J Boirie Y Bruyère O Cederholm T . Sarcopenia: revised European consensus on definition and diagnosis. Age Ageing. (2019) 48:16–31. doi: 10.1093/ageing/afy169 30312372 PMC6322506

[22] Kim DH Rockwood K. Frailty in older adults. N Engl J Med. (2024) 391:538–48. doi: 10.1056/NEJMra2301292 39115063 PMC11634188

[23] Xu W Cai J Liu Y Li L Ye X Wang P . Sarcopenia and frailty among older Chinese adults: Findings from the CHARLS study. PLoS ONE. (2024) 19:e0312879. doi: 10.1371/journal.pone.0312879 39509449 PMC11542859

[24] Beaudart C Demonceau C Reginster JY Locquet M Cesari M Cruz Jentoft AJ . Sarcopenia and health-related quality of life: a systematic review and meta-analysis. J Cachexia Sarcopenia Muscle. (2023) 14:1228–43. doi: 10.1002/jcsm.13243 37139947 PMC10235892

[25] Xu J Wan CS Ktoris K Reijnierse EM Maier AB. Sarcopenia is associated with mortality in adults: a systematic review and meta-analysis. Gerontology. (2022) 68:361–76. doi: 10.1159/000517099 34315158

[26] Goates S Du K Arensberg MB Gaillard T Guralnik J Pereira SL. Economic impact of hospitalizations in US adults with sarcopenia. J Frailty Aging. (2019) 8:93–9. doi: 10.14283/jfa.2019.10 30997923 PMC12275775

[27] Robinson SM. Improving nutrition to support healthy ageing: what are the opportunities for intervention? Proc Nutr Soc. (2018) 77:257–64. doi: 10.1017/S0029665117004037 29173227 PMC6064642

[28] Micek A Błaszczyk-Bebenek E Cebula A Godos J Konopka K Waż A . The bidirectional association of malnutrition with depression and anxiety in patients with cancer: a systematic review and meta-analysis of evidence. Aging Clin Exp Res. (2025) 37:162. doi: 10.1007/s40520-025-03071-y 40410541 PMC12102140

[29] Rasheed S Woods RT. Malnutrition and quality of life in older people: a systematic review and meta-analysis. Ageing Res Rev. (2013) 12:561–6. doi: 10.1016/j.arr.2012.11.003 23228882

[30] Boss GR Seegmiller JE. Age-related physiological changes and their clinical significance. West J Med. (1981) 135:434–40. 7336713 PMC1273316

[31] Donini LM Dominguez LJ Barbagallo M Savina C Castellaneta E Cucinotta D . Senile anorexia in different geriatric settings in Italy. J Nutr Health Aging. (2011) 15:775–81. doi: 10.1007/s12603-011-0048-y 22089227 PMC12878105

[32] Norman K Haß U Pirlich M. Malnutrition in older adults-recent advances and remaining challenges. Nutrients. (2021) 13:2764. doi: 10.3390/nu13082764 34444924 PMC8399049

[33] Mareschal J Genton L Collet TH Graf C. Nutritional intervention to prevent the functional decline in community-dwelling older adults: a systematic review. Nutrients. (2020) 12:9. doi: 10.3390/nu12092820 32942634 PMC7551991

[34] Gielen E Beckwée D Delaere A De Breucker S Vandewoude M Bautmans I . Nutritional interventions to improve muscle mass, muscle strength, and physical performance in older people: an umbrella review of systematic reviews and meta-analyses. Nutr Rev. (2021) 79:121–47. doi: 10.1093/nutrit/nuaa011 32483625

[35] Tessier A Wang F Korat A. Optimal dietary patterns for healthy aging. Nat Med. (2025) 31:1644–52. doi: 10.1038/s41591-025-03570-5 40128348 PMC12092270

[36] Kidney Disease: Improving Global Outcomes (KDIGO) CKD Work Group. KDIGO 2024 clinical practice guideline for the evaluation and management of chronic kidney disease. Kidney Int. (2024) 105:S117–314. doi: 10.1016/j.kint.2023.10.018 38490803

[37] LeRoith D Biessels GJ Braithwaite SS Casanueva FF Draznin B Halter JB . Treatment of diabetes in older adults: an endocrine society^*^ clinical practice guideline. J Clin Endocrinol Metab. (2019) 104:1520–74. doi: 10.1210/jc.2019-00198 30903688 PMC7271968

[38] Wu SY Yeh NH Chang HY Wang CF Hung SY Wu SJ . Adequate protein intake in older adults in the context of frailty: cross-sectional results of the nutrition and health survey in Taiwan 2014–2017. Am J Clin Nutr. (2021) 114:649–60. doi: 10.1093/ajcn/nqab070 33851197

[39] Suresh A Shobna Salaria M Morya S Khalid W Afzal FA . Dietary fiber: an unmatched food component for sustainable health. Food Agri Immunol. (2024) 35:2384420. doi: 10.1080/09540105.2024.2384420

[40] Pérez-López P López-Gómez JJ Izaola-Jauregui O González-Gutiérrez J Estévez-Asensio L Pérez-Mellen I . Relationship between oral intake and sarcopenia in patients with disease-related malnutrition. Nutrients. (2025) 17:13. doi: 10.3390/nu17132129 40647234 PMC12251762

[41] Dinu M Pagliai G Casini A Sofi F. Mediterranean diet and multiple health outcomes: an umbrella review of meta-analyses of observational studies and randomised trials. Eur J Clin Nutr. (2018) 72:30–43. doi: 10.1038/ejcn.2017.58 28488692

[42] ter Borg S Verlaan S Hemsworth J Mijnarends DM Schols JMGA Luiking YC . Micronutrient intakes and potential inadequacies of community-dwelling older adults: a systematic review. Br J Nutr. (2015) 113:1195–206. doi: 10.1017/S0007114515000203 25822905 PMC4531469

[43] Dewiasty E Agustina R Saldi SRF Pramudita A Hinssen F Kumaheri M . Malnutrition prevalence and nutrient intakes of Indonesian community-dwelling older adults: a systematic review of observational studies. Front Nutr. (2022) 9:780003. doi: 10.3389/fnut.2022.780003 35284453 PMC8912970

[44] Mitsopoulou AV Magriplis E Michas G Micha R Chourdakis M Chrousos GP . Micronutrient dietary intakes and their food sources in adults: the hellenic national nutrition and health survey (HNNHS). J Hum Nutr Diet. (2021) 34:616–28. doi: 10.1111/jhn.12840 33497494

[45] Yeung SSY Kwan M Woo J. Composition of healthy diets for older persons. Curr Opin Clin Nutr Metab Care. (2024) 27:17–23. doi: 10.1097/MCO.0000000000000972 37522819

[46] ter Borg S Verlaan S Hemsworth J Mijnarends DM Schols JMGA Luiking YC . Micronutrient intakes and potential inadequacies of community-dwelling older adults: a systematic review. Br J Nutr. (2015) 113:1195–206. doi: 10.1017/S0007114515000203 25822905 PMC4531469

[47] Bandayrel K Wong S. Systematic literature review of randomized control trials assessing the effectiveness of nutrition interventions in community-dwelling older adults. J Nutr Educ Behav. (2011) 43:251–62. doi: 10.1016/j.jneb.2010.01.004 21371944

[48] Pérez-Castillo ÍM Rueda R Pereira SL Bouzamondo H López-Chicharro J Segura-Ortiz F . Age-related anabolic resistance: nutritional and exercise strategies, and potential relevance to life-long exercisers. Nutrients. (2025) 17:3503. doi: 10.3390/nu17223503 41305554 PMC12655298

[49] Paulussen KJM McKenna CF Beals JW Wilund KR Salvador AF Burd NA. Anabolic resistance of muscle protein turnover comes in various shapes and sizes. Front Nutr. (2021) 8:615849. doi: 10.3389/fnut.2021.615849 34026802 PMC8131552

[50] He W Connolly ED Cross HR Wu G. Dietary protein and amino acid intakes for mitigating sarcopenia in humans. Crit Rev Food Sci Nutr. (2025) 65:2538–61. doi: 10.1080/10408398.2024.2348549 38803274

[51] Kim JE O'Connor LE Sands LP Slebodnik MB Campbell WW. Effects of dietary protein intake on body composition changes after weight loss in older adults: a systematic review and meta-analysis. Nutr Rev. (2016) 74:210–24. doi: 10.1093/nutrit/nuv065 26883880 PMC4892287

[52] Campbell WW Deutz NEP Volpi E Apovian CM. Nutritional interventions: dietary protein needs and influences on skeletal muscle of older adults. J Gerontol A Biol Sci Med Sci. (2023) 78:67–72. doi: 10.1093/gerona/glad038 37325954 PMC10272976

[53] Murphy CH McCarthy SN Roche HM. Nutrition strategies to counteract sarcopenia: a focus on protein, LC n-3 PUFA and precision nutrition. Proc Nutr Soc. (2023) 82:419–31. doi: 10.1017/S0029665123003555 37458175

[54] Calvez J Azzout-Marniche D Tomé D. Protein quality, nutrition and health. Front Nutr. (2024) 11:1406618. doi: 10.3389/fnut.2024.1406618 38863590 PMC11165183

[55] Baum JI Wolfe RR. The link between dietary protein intake, skeletal muscle function and health in older adults. Healthcare. (2015) 3:529–43. doi: 10.3390/healthcare3030529 27417778 PMC4939566

[56] Berrazaga I Micard V Gueugneau M Walrand S. The role of the anabolic properties of plant- versus animal-based protein sources in supporting muscle mass maintenance: a critical review. Nutrients. (2019) 11:1825. doi: 10.3390/nu11081825 31394788 PMC6723444

[57] Shams-White MM Chung M Fu Z Insogna KL Karlsen MC LeBoff MS . Animal versus plant protein and adult bone health: a systematic review and meta-analysis from the national osteoporosis foundation. PLoS ONE. (2018) 13:e0192459. doi: 10.1371/journal.pone.0192459 29474360 PMC5825010

[58] Reid-McCann RJ Brennan SF McKinley MC McEvoy CT. The effect of animal versus plant protein on muscle mass, muscle strength, physical performance and sarcopenia in adults: protocol for a systematic review. Syst Rev. (2022) 11:64. doi: 10.1186/s13643-022-01951-2 35418173 PMC9006591

[59] Paddon-Jones D Campbell WW Jacques PF Kritchevsky SB Moore LL Rodriguez NR . Protein and healthy aging. Am J Clin Nutr. (2015) 101:1339S−45S. doi: 10.3945/ajcn.114.084061 25926511

[60] Reid-McCann RJ Brennan SF Ward NA Logan D McKinley MC McEvoy CT. Effect of plant versus animal protein on muscle mass, strength, physical performance, and sarcopenia: a systematic review and meta-analysis of randomized controlled trials. Nutr Rev. (2025) 83:e1581–603. doi: 10.1093/nutrit/nuae200 39813010 PMC12166177

[61] Basisty N Meyer JG Schilling B. Protein turnover in aging and longevity. Proteomics. (2018) 18:e1700108. doi: 10.1002/pmic.201700108 29453826 PMC6022828

[62] Dominique D Mosoni L Savary-Auzeloux I Peyron MA Polakof S Rémond D. Important determinants to take into account to optimize protein nutrition in the elderly: solutions to a complex equation. Proc Nutr Soc. (2021) 80:207–20. doi: 10.1017/S0029665120007934 33198824

[63] Li CWD Herpich C Haß U Kochlik B Weber D Grune T . Essential amino acids and branched-chain amino acids are associated with skeletal muscle and inflammatory parameters in older age. Biogerontology. (2025) 26:66. doi: 10.1007/s10522-025-10206-1 40045114 PMC11882671

[64] Bougel C Servien R Vialaneix N Maigne E Boirie Y Lahaye C . Serum metabolomic signatures associated with frailty-related phenotypes in a cohort of older people. Geroscience. (2026). doi: 10.1007/s11357-026-02116-y. [Epub ahead of print]. 41619134 PMC13638843

[65] Golshany H Helmy SA Morsy NFS Kamal A Yu Q Fan L. The gut microbiome across the lifespan: how diet modulates our microbial ecosystem from infancy to the elderly. Int J Food Sci Nutr. (2025) 76:95–121. doi: 10.1080/09637486.2024.2437472 39701663

[66] Coelho-Junior HJ Marzetti E Picca A Cesari M Uchida MC Calvani R. Protein intake and frailty: a matter of quantity, quality, and timing. Nutrients. (2020) 12:10. doi: 10.3390/nu12102915 32977714 PMC7598653

[67] Bai GH Tsai MC Tsai HW Chang CC Hou WH. Effects of branched-chain amino acid-rich supplementation on EWGSOP2 criteria for sarcopenia in older adults: a systematic review and meta-analysis. Eur J Nutr. (2022) 61:637–51. doi: 10.1007/s00394-021-02710-0 34705076

[68] Cochet C Belloni G Buondonno I Chiara F D'Amelio P. The role of nutrition in the treatment of sarcopenia in old patients: from restoration of mitochondrial activity to improvement of muscle performance, a systematic review. Nutrients. (2023) 15:17. doi: 10.3390/nu15173703 37686735 PMC10490489

[69] Park S Chae M Park H Park K. Higher branched-chain amino acid intake is associated with handgrip strength among Korean older adults. Nutrients. (2021) 13:1522. doi: 10.3390/nu13051522 33946360 PMC8146867

[70] Mantuano P Boccanegra B Bianchini G Cappellari O Tulimiero L Conte E . Branched-chain amino acids and di-alanine supplementation in aged mice: a translational study on sarcopenia. Nutrients. (2023) 15:2. doi: 10.3390/nu15020330 36678201 PMC9861351

[71] Ruiz-Pozo V Guevara P Paz Cruz E Tamayo Trujillo R Cadena-Ullauri S Frias-Toral E . The role of the Mediterranean diet in prediabetes management and prevention: a review of molecular mechanisms and clinical outcomes. Food Agri Immunol. (2024) 35:2398042. doi: 10.1080/09540105.2024.2398042

[72] Liu H Zhang Q Hao Q Li Q Yang L Yang X . Associations between sarcopenia and circulating branched-chain amino acids: a cross-sectional study over 100,000 participants. BMC Geriatr. (2024) 24:541. doi: 10.1186/s12877-024-05144-5 38907227 PMC11193178

[73] Zhu X Wang J Lu Y Zhao Y Zhang N Wu W . Potential of food protein-derived bioactive peptides against sarcopenia: a comprehensive review. J Agric Food Chem. (2023) 71:5419–37. doi: 10.1021/acs.jafc.2c09094 36988097

[74] Caldo-Silva A Furtado GE Chupel MU Letieri RV Neves RS Direto F . Empowering frail older adults: multicomponent elastic-band exercises and BCAA supplementation unleash physical health and preserve haematological biomarkers. Front Sports Act Living. (2023) 5:1171220. doi: 10.3389/fspor.2023.1171220 37720080 PMC10502309

[75] Zhao Y Cholewa J Shang H Yang Y Ding X Wang Q . Advances in the role of leucine-sensing in the regulation of protein synthesis in aging skeletal muscle. Front Cell Dev Biol. (2021) 9:646482. doi: 10.3389/fcell.2021.646482 33869199 PMC8047301

[76] Casperson SL Sheffield-Moore M Hewlings SJ Paddon-Jones D. Leucine supplementation chronically improves muscle protein synthesis in older adults consuming the RDA for protein. Clin Nutr. (2012) 31:512–9. doi: 10.1016/j.clnu.2012.01.005 22357161 PMC3640444

[77] Lee JH Jeon JH Lee MJ. Docosahexaenoic acid, a potential treatment for sarcopenia, modulates the ubiquitin–proteasome and the autophagy–lysosome systems. Nutrients. (2020) 12:9. doi: 10.3390/nu12092597 32859116 PMC7551806

[78] Chen S Chen J Wang C He T Yang Z Huang W . Betaine attenuates age-related suppression in autophagy via Mettl21c/p97/VCP axis to delay muscle loss. J Nutr Biochem. (2024) 125:109555. doi: 10.1016/j.jnutbio.2023.109555 38147913

[79] Ballesteros J Rivas D Duque G. The role of the kynurenine pathway in the pathophysiology of frailty, sarcopenia, and osteoporosis. Nutrients. (2023) 15:14. doi: 10.3390/nu15143132 37513550 PMC10383689

[80] Ali SR Nkembo AT Tipparaju SM Ashraf M Xuan W. Sarcopenia: recent advances for detection, progression, and metabolic alterations along with therapeutic targets. Can J Physiol Pharmacol. (2024) 102:697–708. doi: 10.1139/cjpp-2024-0201 39186818 PMC11663012

[81] Dukes A Davis C El Refaey M Upadhyay S Mork S Arounleut P . The aromatic amino acid tryptophan stimulates skeletal muscle IGF1/p70s6k/mTor signaling in vivo and the expression of myogenic genes in vitro. Nutrition. (2015) 31:1018–24. doi: 10.1016/j.nut.2015.02.011 26059377 PMC4465076

[82] Kaiser H Yu K Pandya C Mendhe B Isales CM McGee-Lawrence ME . Kynurenine, a tryptophan metabolite that increases with age, induces muscle atrophy and lipid peroxidation. Oxid Med Cell Longev. (2019) 2019:9894238. doi: 10.1155/2019/9894238 31737181 PMC6815546

[83] Ninomiya S Nakamura N Nakamura H Mizutani T Kaneda Y Yamaguchi K . Low levels of serum tryptophan underlie skeletal muscle atrophy. Nutrients. (2020) 12:4. doi: 10.3390/nu12040978 32244785 PMC7230402

[84] Yang Y Pan N Luo J Liu Y Ossowski Z. Exercise and nutrition for sarcopenia: a systematic review and meta-analysis with subgroup analysis by population characteristics. Nutrients. (2025) 17:2342. doi: 10.3390/nu17142342 40732966 PMC12299059

[85] Olaniyi KS Olatunji LA. Inhibition of pyruvate dehydrogenase kinase-4 by l-glutamine protects pregnant rats against fructose-induced obesity and hepatic lipid accumulation. Biomed Pharmacother. (2019) 110:59–67. doi: 10.1016/j.biopha.2018.11.038 30466003

[86] He Y Song Z Ji Y Tso P Wu Z. Preventive effects of l-glutamine on high-fat diet-induced metabolic disorders linking with regulation of intestinal barrier integrity, hepatic lipid metabolism, and gut microbiota in rats. J Agric Food Chem. (2022) 70:11923–34. doi: 10.1021/acs.jafc.2c01975 36122193

[87] Olaniyi KS Olatunji LA. L-glutamine ameliorates adipose-hepatic dysmetabolism in OC-treated female rats. J Endocrinol. (2020) 246:1–12. doi: 10.1530/JOE-19-0582 32413841

[88] Lai JC Tandon P Bernal W Tapper EB Ekong U Dasarathy S . Malnutrition, frailty, and sarcopenia in patients with cirrhosis: 2021 practice guidance by the American association for the study of liver diseases. Hepatology. (2021) 74:1611–44. doi: 10.1002/hep.32049 34233031 PMC9134787

[89] Dasarathy S Merli M. Sarcopenia from mechanism to diagnosis and treatment in liver disease. J Hepatol. (2016) 65:1232–44. doi: 10.1016/j.jhep.2016.07.040 27515775 PMC5116259

[90] Olaniyi KS Sabinari IW Olatunji LA. Oral L-glutamine restores adenosine and glutathione content in the skeletal muscle and adipose tissue of insulin-resistant pregnant rats. Nutrition. (2020) 77:110789. doi: 10.1016/j.nut.2020.110789 32428839

[91] Liu N Ma X Luo X Zhang Y He Y Dai Z . l-glutamine attenuates apoptosis in porcine enterocytes by regulating glutathione-related redox homeostasis. J Nutr. (2018) 148:526–34. doi: 10.1093/jn/nxx062 29659951

[92] Meynial-Denis D. Glutamine metabolism in advanced age. Nutr Rev. (2016) 74:225–36. doi: 10.1093/nutrit/nuv052 26936258 PMC4892310

[93] Coelho CRF. Effect of glutamine supplementation in elderly people with emphasis on sarcopenia, immunity and hypertrophy: a concise systematic review. Int J Nutrol. (2023) 16:ijn23108. doi: 10.54448/ijn23108

[94] Matsuyama T Okada H Saijo Y Hasegawa Y Nakajima H Okamura T . Effects of vitamin D intake alone on elderly body composition: a systematic review and meta-analysis of RCTs. Arch Gerontol Geriatr. (2026) 142:106093. doi: 10.1016/j.archger.2025.106093 41319491

[95] Díaz P Cadena M Montalván ME Garrochamba K Calderón P Carrión G . Hypovitaminosis D in university workers in Southern Ecuador: interactions between gender and lifestyle. Front Nutr. (2024) 11:1482910. doi: 10.3389/fnut.2024.1482910 39391680 PMC11464991

[96] Kreider RB Stout JR. Creatine in health and disease. Nutrients. (2021) 13:447. doi: 10.3390/nu13020447 33572884 PMC7910963

[97] Greenhaff PL. The creatine-phosphocreatine system: there's more than one song in its repertoire. J Physiol. (2001) 537:657. doi: 10.1111/j.1469-7793.2001.00657.x 11744744 PMC2278982

[98] Candow DG Forbes SC Chilibeck PD Cornish SM Antonio J Kreider RB. Effectiveness of creatine supplementation on aging muscle and bone: focus on falls prevention and inflammation. J Clin Med. (2019) 8:488. doi: 10.3390/jcm8040488 30978926 PMC6518405

[99] Liu S Zhang L Li S. Advances in nutritional supplementation for sarcopenia management. Front Nutr. (2023) 10:1189522. doi: 10.3389/fnut.2023.1189522 37492597 PMC10365293

[100] Forbes SC Candow DG Ostojic SM Roberts MD Chilibeck PD. Meta-analysis examining the importance of creatine ingestion strategies on lean tissue mass and strength in older adults. Nutrients. (2021) 13:6. doi: 10.3390/nu13061912 34199420 PMC8229907

[101] Candow DG Chilibeck PD Forbes SC Fairman CM Gualano B Roschel H. Creatine supplementation for older adults: focus on sarcopenia, osteoporosis, frailty and cachexia. Bone. (2022) 162:116467. doi: 10.1016/j.bone.2022.116467 35688360

[102] Ravindran R Pizzol D López-Gil JF Rahmati M Boyer L Fond G . Collagen supplementation for skin and musculoskeletal health: an umbrella review of meta-analyses on elasticity, hydration, and structural outcomes. Aesthet Surg J Open Forum. (2026) 8:ojag018. doi: 10.1093/asjof/ojag018 41809116 PMC12968778

[103] Bischof K Moitzi AM Stafilidis S König D. Impact of collagen peptide supplementation in combination with long-term physical training on strength, musculotendinous remodeling, functional recovery, and body composition in healthy adults: a systematic review with meta-analysis. Sports Med. (2024) 54:2865–88. doi: 10.1007/s40279-024-02079-0 39060741 PMC11561013

[104] Ivaskiene T Viskelis J Streimikyte P Savickaite M Mobasheri A Kaspute G. Collagen supplementation and regenerative health: advances in biomarker detection and smart material integration. Front Nutr. (2025) 12:1716166. doi: 10.3389/fnut.2025.1716166 41459089 PMC12739960

[105] Porfírio E Fanaro GB. Collagen supplementation as a complementary therapy for the prevention and treatment of osteoporosis and osteoarthritis: a systematic review. Rev bras geriatr gerontol. (2016) 19:153–64. doi: 10.1590/1809-9823.2016.14145

[106] Zdzieblik D Oesser S Baumstark MW Gollhofer A König D. Collagen peptide supplementation in combination with resistance training improves body composition and increases muscle strength in elderly sarcopenic men: a randomised controlled trial. Br J Nutr. (2015) 114:1237–45. doi: 10.1017/S0007114515002810 26353786 PMC4594048

[107] Oikawa SY McGlory C D'Souza LK Morgan AK Saddler NI Baker SK . A randomized controlled trial of the impact of protein supplementation on leg lean mass and integrated muscle protein synthesis during inactivity and energy restriction in older persons. Am J Clin Nutr. (2018) 108:1060–8. doi: 10.1093/ajcn/nqy193 30289425

[108] Zhang S Miller DD Li W. Non-musculoskeletal benefits of vitamin D beyond the musculoskeletal system. Int J Mol Sci. (2021) 22:2128. doi: 10.3390/ijms22042128 33669918 PMC7924658

[109] Voiculescu VM Nelson Twakor A Jerpelea N Pantea Stoian A. Vitamin D: beyond traditional roles-insights into its biochemical pathways and physiological impacts. Nutrients. (2025) 17:803. doi: 10.3390/nu17050803 40077673 PMC11902150

[110] Zhang F Li W. Vitamin D and sarcopenia in the senior people: a review of mechanisms and comprehensive prevention and treatment strategies. TCRM. (2024) 20:577–95. doi: 10.2147/TCRM.S471191 39253031 PMC11382659

[111] Paiva AJH de Santos GF Ferreira LP Pereira AC de S Avellar AP Coutinho BT . Effects of vitamin D supplementation in sarcopenic elderly: an integrative review. Res Soc Dev. (2022) 11:12. doi: 10.33448/rsd-v11i12.35090

[112] Frazer EJ Price RK Rosbotham EJ Royle E Hollywood L Pourshahidi LK. Global trends in vitamin D-fortified food and drink product launches (2019–2023). Int J Food Sci Nutr. (2025) 76:689–700. doi: 10.1080/09637486.2025.2567943 41084220

[113] Sakuma K Hamada K Yamaguchi A Aoi W. Current nutritional and pharmacological approaches for attenuating sarcopenia. Cells. (2023) 12:19. doi: 10.3390/cells12192422 37830636 PMC10572610

[114] Widajanti N Hadi U Soelistijo SA Syakdiyah NH Rosaudyn R Putra HBP. The effect of vitamin D supplementation to parameter of sarcopenia in elderly people: a systematic review and meta-analysis. Can Geriatr J. (2024) 27:63–75. doi: 10.5770/cgj.27.694 38433884 PMC10896205

[115] Ferguson EJ Seigel JW McGlory C. Omega-3 fatty acids and human skeletal muscle. Curr Opin Clin Nutr Metab Care. (2021) 24:114–9. doi: 10.1097/MCO.0000000000000723 33332930

[116] Chicaiza CDN Veloz SEB. Protein supplementation in older adults, a way to prevent sarcopenia. BIOSANA Sci Health Magazine. (2025) 5:2. doi: 10.62305/biosana.v5i2.485

[117] Liang Z Zhang L. Chronic inflammation as a driving factor for sarcopenia: an update on pathophysiology and future therapeutic targets. Front Pharmacol. (2026) 17:1733798. doi: 10.3389/fphar.2026.1733798 41808874 PMC12968189

[118] Yoshino J Smith GI Kelly SC Julliand S Reeds DN Mittendorfer B. Effect of dietary n-3 PUFA supplementation on the muscle transcriptome in older adults. Physiol Rep. (2016) 4:e12785. doi: 10.14814/phy2.12785 27252251 PMC4908485

[119] Zhang Y Guo H Liang J Xiao W Li Y. Relationship between dietary omega-3 and omega-6 polyunsaturated fatty acids level and sarcopenia. a meta-analysis of observational studies. Front Nutr. (2022) 8:738083. doi: 10.3389/fnut.2021.738083 35096921 PMC8789889

[120] Gutierres D Pacheco R Reis CP. The role of omega-3 and omega-6 polyunsaturated fatty acid supplementation in human health. Foods. (2025) 14:3299. doi: 10.3390/foods14193299 41097470 PMC12524211

[121] Mansoor U Edano D Usman M Habib U. The impact of nutritional supplements on sarcopenia: a systematic review and meta-analysis. Cureus. (2025) 17:e88459. doi: 10.7759/cureus.88459 40842757 PMC12367323

[122] Ye L Liang R Liu X Li J Yue J Zhang X. Frailty and sarcopenia: a bibliometric analysis of their association and potential targets for intervention. Ageing Res Rev. (2023) 92:102111. doi: 10.1016/j.arr.2023.102111 38031836

[123] Li Y Liu Z Yan H Zhou T Zheng L Wen F . Polygonatum sibiricum polysaccharide ameliorates skeletal muscle aging and mitochondrial dysfunction via PI3K/Akt/mTOR signaling pathway. Phytomedicine. (2025) 136:156316. doi: 10.1016/j.phymed.2024.156316 39674120

[124] Losasso MR Parussolo MLC Oliveira Silva A Direito R Quesada K Penteado Detregiachi CR . Unraveling the metabolic pathways between metabolic-associated fatty liver disease (MAFLD) and sarcopenia. Int J Mol Sci. (2025) 26:10. doi: 10.3390/ijms26104673 40429815 PMC12111209

[125] Portal ICBF - Instituto Colombiano de Bienestar Familiar ICBF. Tabla de Composición de Alimentos Colombianos. Available online at: https://www.icbf.gov.co/bienestar/nutricion/tabla-alimentos (Accessed August 05, 2025).

[126] de Miranda RB Weimer P Rossi RC. Effects of hydrolyzed collagen supplementation on skin aging: a systematic review and meta-analysis. Int J Dermatol. (2021) 60:1449–61. doi: 10.1111/ijd.15518 33742704

[127] Dupont J Dedeyne L Dalle S Koppo K Gielen E. The role of omega-3 in the prevention and treatment of sarcopenia. Aging Clin Exp Res. (2019) 31:825–36. doi: 10.1007/s40520-019-01146-1 30784011 PMC6583677

[128] Kakehi S Wakabayashi H Inuma H Inose T Shioya M Aoyama Y . Rehabilitation nutrition and exercise therapy for sarcopenia. World J Mens Health. (2022) 40:1–10. doi: 10.5534/wjmh.200190 33831974 PMC8761238

